# Identification of Candidate Salivary, Urinary and Serum Metabolic Biomarkers for High Litter Size Potential in Sows (*Sus scrofa*)

**DOI:** 10.3390/metabo12111045

**Published:** 2022-10-30

**Authors:** Lauren Fletcher, Nadeem Akhtar, Xiaoshu Zhan, Mohsen Jafarikia, Brian P. Sullivan, Lee-Anne Huber, Julang Li

**Affiliations:** 1 Department of Animal Biosciences, University of Guelph, Guelph, ON N1G 2W1, Canada; 2 Department of Life Science and Engineering, Foshan University, Foshan 528231, China; 3 Canadian Centre for Swine Improvement Inc., Ottawa, ON K1A 0C6, Canada

**Keywords:** metabolomics, infertility, reproductive potential, litter size, serum, urine, saliva, gilt selection, LC-MS/MS

## Abstract

The selection of sows that are reproductively fit and produce large litters of piglets is imperative for success in the pork industry. Currently, low heritability of reproductive and litter-related traits and unfavourable genetic correlations are slowing the improvement of pig selection efficiency. The integration of biomarkers as a supplement or alternative to the use of genetic markers may permit the optimization and increase of selection protocol efficiency. Metabolite biomarkers are an advantageous class of biomarkers that can facilitate the identification of cellular processes implicated in reproductive condition. Metabolism and metabolic biomarkers have been previously implicated in studies of female mammalian fertility, however a systematic analysis across multiple biofluids in infertile and high reproductive potential phenotypes has not been explored. In the current study, the serum, urinary and salivary metabolomes of infertile (INF) sows and high reproductive potential (HRP) sows with a live litter size ≥ 13 piglets were examined using LC-MS/MS techniques, and a data pipeline was used to highlight possible metabolite reproductive biomarkers discriminating the reproductive groups. The metabolomes of HRP and INF sows were distinct, including significant alterations in amino acid, fatty acid, membrane lipid and steroid hormone metabolism. Carnitines and fatty acid related metabolites were most discriminatory in separating and classifying the HRP and INF sows based on their biofluid metabolome. It appears that urine is a superior biofluid than saliva and serum for potentially predicting the reproductive potential level of a given female pig based on the performance of the resultant biomarker models. This study lays the groundwork for improving gilt and sow selection protocols using metabolomics as a tool for the prediction of reproductive potential.

## 1. Introduction

The efficient selection of gilts and sows for the breeding herd is a vital step in the pork farming process, affecting overall production of the operation and economic returns [1,2]. Due to the influence of genetics on pig performance, the examination of maternal lines for genetically superior sows is commonly integrated into selection protocols [3,4]. This allows for accurate selection and swift genetic progress in production traits, but when it comes to reproductive traits related to the litter, methods of selection using solely genetic markers for selection are not highly efficient [5]. Critical litter related traits, including mean litter size, number of live-born piglets, number of stillborn piglets and number of piglets alive at weaning do not have substantial heritability [6,7]. In addition, there are negative genetic correlations that exist between production traits and reproductive traits, such as age at 100 kg with number of piglets born alive per litter, resulting in unfavourable selection tradeoffs [8]. These genetic roadblocks to selection for reproductive performance leads to slower genetic progress, the over-selection of gilts to meet production requirements, high culling rates, and lower economic returns [9]. Moreover, pigs are unique in comparison to other domestic livestock, suffering from higher rates of pregnancy failure and infertility due to embryonic mortality, intrauterine growth restriction and neonatal deaths [10,11,12]. A more efficient method to supplement the use of genetic markers in gilt and sow selection protocols to better select for high reproducers would improve welfare by reducing the number of gilts in the development pool, production by increasing number of piglets born, and overall economic returns of operations in the pork industry.

Metabolomics is an emerging investigative tool that offers a more efficient alternative to circumvent the challenges and limitations facing current genetic-based gilt and sow selection. The state of cellular processes and metabolism reflect specific genomic, proteomic, physiologic and microenvironmental differences between individuals [13,14,15]. Metabolomics allows for the identification of these subtle cellular processes and metabolism differences [16,17,18]. In studies of human fertility, metabolomics and biomarker identification is becoming a popular and effective tool, with studies characterizing the metabolic phenotypes of reproductive conditions including polycystic ovary syndrome (PCOS) [19,20,21,22,23,24,25] (reviewed in [26]), primary ovary insufficiency (POI) [27], poor oocyte and embryo quality [28], and uterine conditions including endometriosis, adenomyosis and cancer (reviewed in [29]). In the characterization of these phenotypes metabolites involved in amino acid, fatty acid, lipid and steroid hormone metabolism continuously appear as important biomarkers [19,20,21,22,23,24,25,26,27,28,29]. Based on the results of these studies, it is possible that the targeting metabolomic evaluation of fluids isolated from sows for similar metabolic pathways could provide a more in-depth and comprehensive view of their reproductive state, and provide biomarker information for making selection decisions.

To date, two studies have been performed in characterizing sows of low oocyte quality and normal or high oocyte quality using follicular fluid, urine and serum, citing disruptions in glucose, amino acid, fatty acid and purine metabolism and oxidative stress in poor oocyte quality [30,31]. However, the comparison of high reproductive potential and infertile female metabolomes in any mammal has not been reported, and a large sample size is needed to accurately assess the relevance of biomarkers for selection or identification of reproductive potential level. The overall objective of the study was to investigate the possibility of using metabolomics to characterize high reproductive potential and infertile female pig phenotypes and identify metabolite biomarkers in urine, saliva and serum biofluids. We also aimed to provide further insight into the amino acid, fatty acid, lipid and steroid hormone metabolic pathways that have been implicated in fertility, contributing the understanding surrounding the interactions between metabolism and fertility.

## 2. Methods

### 2.1. Animals and Housing

All animal procedures were carried out in accordance to the Canadian Council on Animal Care (CCAC) guidelines under the Animal Utilization Protocol (AUP) #4037 approved by Animal Care Services (ACS) at the University of Guelph. A total of 53, 46 and 69 pigs (purebred Yorkshire and Yorkshire × Landrace crosses) were sampled for urine, saliva and serum and were distributed in two groups based on their high or infertile reproductive status. Animals were individually housed in stalls or a group setting with access to water and restricted feed at Arkell Research Station (University of Guelph, Guelph, ON) or Alliance Genetic Canada’s affiliated operations. High reproductive potential (HRP) sows were defined as pigs with ≥13 piglets born alive (NPBA) on average throughout their reproductive lifespan immediately prior to sampling. Infertile sows (INF) were defined as pigs that had failed to conceive after two rounds of back-to-back artificial insemination just prior to sampling (consecutive estrus cycles).

### 2.2. Biofluid Collection

To limit metabolic profile variability stemming from reproductive cycle stage [32], samples were collected from estrus-synchronized pigs. Sows were sampled four to five days after weaning which is the time point or commencement of the estrus cycle in sows [32]. To limit circadian and diurnal variation, samples were collected from pigs in the morning, in the same two-hour time window.

Saliva was collected using the cable-lock-assisted swab method outlined in Akhtar et al. [33]. Briefly, Salivette^®^ tubes with plain cotton swabs (Starstedt AG & Co.), specialized for collection and processing methods were used to ensure the hygienic sampling of saliva. A plain cotton swab was removed from the tube and attached to the end of a zip-tie and directed towards the buccal cavity of the pig. Pigs were permitted to chew on the swab for 30 s to ensure sufficient saturation from the salivary glands of the buccal cavity. The swab was removed from the zip-tie and placed back into the Salivette^®^ tubes. Tubes were centrifuged at 2000× *g* and 4 °C for 5 min. Saliva was aliquoted and stored at −80 °C until metabolomic analyses. 

Urine was obtained using a tampon collection method [34]. Briefly, an unscented tampon was inserted into the pig’s vestibule and the string was secured to the rump using waterproof tape. Pigs were monitored for urination. Immediately after urination and saturation of the tampon, the tampon was removed and urine was sqeezed from the tampn with clean gloves to a sterile falcon tube. Samples were left undisturbed for 30 min (allowing for the particulates to settle) and the supernatant was aliquoted and stored at −80 °C until metabolomic analyses. 

Blood collection was performed through orbital sinus bleeding as outlined by Dove and Alworth [35]. Briefly, BD PrecisionGlideTM General Use Sterile Hypodermic Needles (22-gauge, 3.8 cm) were used to puncture the orbital sinus at the medial canthus. Blood was collected into sterile falcon tubes and left undisturbed and upright for 1 h at room temperature to clot. Once clotted, the samples were centrifuged (2000× *g*, 10 min, 21 °C) and the resulting supernatant (serum) was aliquoted and stored at −80 °C until metabolomic analyses.

### 2.3. Metabolomic Analysis

Targeted quantitative metabolomic analysis of the biofluid samples using direct injection (DI), liquid chromatography (LC) and mass-spectrometry (MS). Analyses were outsourced to The Metabolomics Innovation Centre (TMIC, University of Alberta, Edmonton, AB, Canada). Mass spectrometric analysis was performed on an API4000 Qtrap^®^ tandem mass spectrometry instrument (Sciex Canada, Concord, ON, Canada) equipped with an Agilent 1260 series HPLC system (Agilent Technologies, Palo Alto, CA, USA). Liquid chromatography coupled with tandem mass spectroscopy (LC-MS/MS) was used to detect steroid hormones, including cortisol, androstenedione, progesterone, androsterone, estrone, testosterone, DHEA, and 17-hydroxyprogesterone. Direct-injection mass spectroscopy with reverse phase liquid chromatography and tandem mass-spectroscopy (DI/LC-MS/MS) was used to analyze the samples for up to 135 endogenous metabolites, including essential and non-essential amino acids, acylcarnitines, biogenic amines and derivatives, uremic toxins, glycerophospholipids, sphingolipids and sugars.

### 2.4. Data Pre-Processing and Statistical Analysis

Data processing and statistical analysis was performed using a data pipeline constructed in the Python programming language (Python Software Foundation. Python Language Reference, Version 3.9.7. Available at: https://www.python.org). Metaboanalyst (Version 5.0, Available at: www.metaboanalyst.ca) was used for construction of the Volcano plots [36]. Raw LC-MS/MS concentration data was pre-processed using quantile normalization, log transformation and pareto scaling to account for dilution differences between samples or measurement variation between samples [37]. Pig samples missing more than 70% of values were excluded from the analysis. Metabolite features missing more than 50% of values were excluded from the analysis, and the missing values of the remaining features were replaced by 1/5 of the LOD (1/5 of the minimum value of each variable). Data is presented as mean ± SEM, unless otherwise indicated. Statistical significance was assumed at *p* ≤ 0.05. Fold-changes > 2 were deemed significant.

### 2.5. Variable Selection and Potential Biomarker Identification

**Figure 1:**
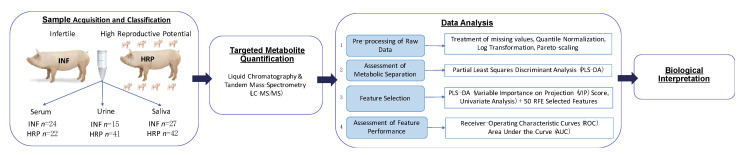
Flow of the study. Pigs were sampled for saliva, urine and serum as outlined in the methods section. After sampling, pigs were classified into their respective group based on their past reproductive performance. Samples were analyzed with targeted metabolomic quantification using LC-MS/MS techniques. Raw data from this analysis was appropriately pre-processed and analyzed using our developed metabolomics protocol. Metabolomics analysis results were then interpreted in the context of biological relevance of sow reproduction.

Variable or feature selection is a method performed before the application of a data set to machine learning or classification models [38]. It reduces the dimensionality of datasets which can pose significant challenges to classification models [38,39,40]. It is particularly important when dealing with the ‘curse of dimensionality’ where the number of features (i.e., metabolites) in a dataset extremely outnumbers the number of records [38]. Feature selection can improve the accuracy of a model and decrease overfitting, as having irrelevant variables can decrease classification model accuracy [38,39]. In the present study, feature selection was completed using a combination of the supervised multivariate method Partial-Least Squares Discriminant Analysis (PLS-DA) and the feature selection algorithm Recursive Feature Elimination (RFE). Cross validation to confirm the validity of the PLS-DA model was performed through the 10-fold repeated cross-validation method, using R2 to measure the proportion of variance explained and model predictability, and mean squared error (MSE) as the average difference between the predicted and actual sample classes. Recursive feature elimination was performed via a Decision Tree Classifier, selecting 50 candidate metabolites and verified with accuracy. Significant (*p* < 0.05) metabolites with a Variable Importance in Projection (VIP) score ≥ 1.25 (indicating a measurable contribution to the separation of reproductive groups based on metabolite level in the PLS-DA model) that were also features selected in the RFE process, were selected for Receiver Operating Characteristic (ROC) diagnostic classifier analysis. The choice for the VIP cutoff value of 1.25 is reviewed in [41]. ROC curves reporting the performance of a Support-Vector Machine (SVM) classifier via stratified K-fold cross validation plotting true positive rate (sensitivity) against false positive rate (100-specificity) at various thresholds were used to analyze the predictive capabilities of the metabolite biomarkers. Area under the curve (AUC), confusion matrices and permutations were used to evaluate the performance of the classifiers. A visual representation of this workflow is seen in the Data Analysis Section of Figure 1.

## 3. Results

The flowchart of the study is outlined in Figure 1. After grouping pigs based on reproductive performance and sample availability, a total of 48 serum (HRP *n* = 22, INF *n* = 24), 56 urine (HRP *n* = 41, INF *n* = 15), and 69 saliva (HRP *n* = 42, INF *n* = 27) samples were included for metabolomics analysis.

### 3.1. HRP and INF Sows Have Diverging Metabolic Profiles in Saliva, Urine and Serum

**Figure 2:**
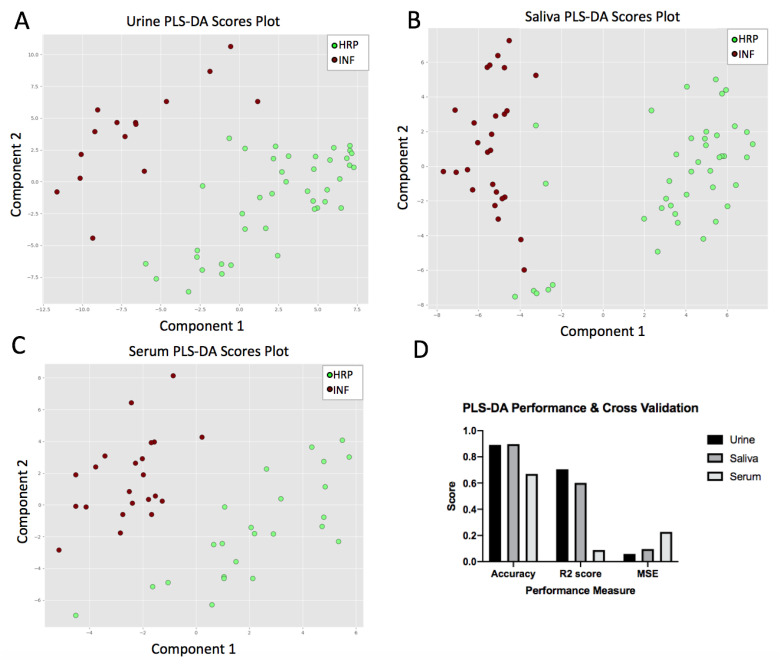
Distinct metabolic separation between HRP and INF pigs based on concentration of analyzed metabolites. PLS-DA plots clearly separated the HRP and INF groups using metabolite concentrations evaluated in urine (**A**), saliva (**B**), and serum (**C**). Cross validation and performance measures (**D**) of the PLS-DA models suggest that urine and saliva perform better than serum in the metabolic separation of the HRP and INF groups.

After metabolomic analysis and data pre-processing, 125, 123 and 115 metabolites were suitable to include in the statistical analysis for serum, urine and saliva, respectively (Supplementary Tables S1–S3). Multivariate PLS-DA analysis was performed to identify metabolic separation between HRP and INF sows and begin the feature selection process. Overall, metabolic separation between the HRP and INF groups was observed in all three biofluid types (Urine; Figure 2A, Saliva; Figure 2B, Serum; Figure 2C). Urine and saliva appeared to have the highest degree of separation in the PLS-DA models, with an average accuracy of 0.89 and 0.90, respectively, moderate predictability (Urine R2 = 0.71, Saliva R2 = 0.60) and acceptable levels of error (Urine MSE = 0.06, Saliva MSE = 0.10). Serum had less separation between groups, with an average accuracy of 0.67, lower predictive ability (R2 = 0.09) and higher error (MSE = 0.23; Figure 2D). Feature selection through Recursive Feature Elimination (RFE) aligned with the PLS-DA feature selection results, with the urine selected features having the highest accuracy (0.929 ± 0.098), followed by the saliva selected features (0.851 ± 0.108) and the serum selected features (0.758 ± 0.204).

### 3.2. Amino Acid, Fatty-Acid, Lipid and Steroid Hormone Metabolites Are Altered between HRP and INF Sows

**Figure 3:**
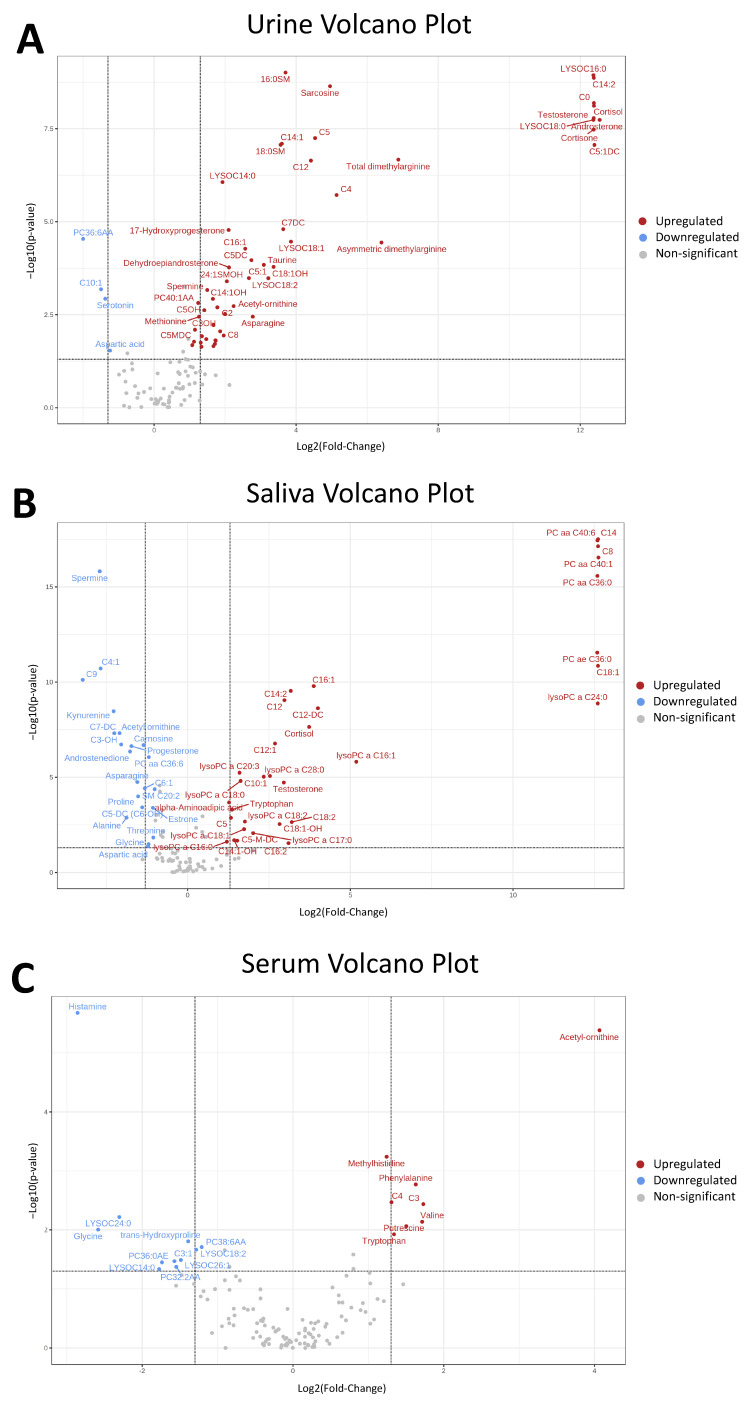
Volcano plots depicting the significant differences of metabolite concentrations in the urine (**A**), saliva (**B**), and serum (**C**) of HRP pigs in comparison to INF pigs. Each dot represents a metabolite. Red metabolites indicate an upregulation of the metabolite in HRP sows in comparison to INF sows, blue metabolites indicate a downregulation of the metabolite in HRP sows in comparison to INF sows, and grey metabolites indicate no change between HRP and INF sows. Fold-Change threshold = 2, *p*-value threshold = 0.05. A full list of *p*-values and FC values can be found in Supplementary Tables S4–S6.

**Figure 4:**
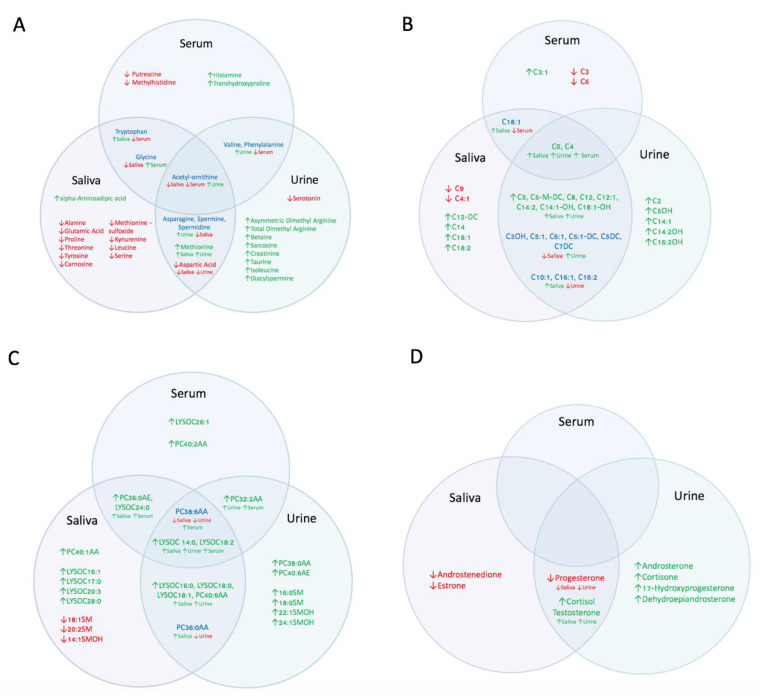
Comparison of the metabolite changes of amino acid (**A**), fatty acid oxidation (**B**), lipid membrane (**C**) and steroid hormone (**D**) metabolite groups in serum, urine and saliva between HRP and INF pigs. Red metabolites indicate a decrease in the concentration of the metabolite in HRP compared to INF, green metabolites indicate an increase in the concentration of the metabolite in HRP compared to INF, and blue metabolites indicate metabolites that have variable changes in the metabolite across more than one biofluid, with the direction of their changes indicated by the arrow and corresponding biofluid underneath.

Univariate and fold-change analysis of all metabolites revealed significant metabolic differences in urine (Figure 3A), saliva (Figure 3B), and serum (Figure 3C) samples when comparing HRP and INF sows. Significant metabolite concentration differences were identified in all metabolite groups targeted, including amino acids/biogenic amines, carnitines, phospholipids and steroid hormones (Figure 4). Interestingly acetyl-ornithine, acetyl carnitine (C0), butrurylcarnitine (C4), two glycerophospholipids (LYSOC 14:0, LYSOC 18:2) and a phosphatidylcholine (PC38:6AA) were found at significantly different concentrations between HRP and INF pigs across all three biofluids (Figure 4A–C).

Of the amino acids and biogenic amine derivatives (Figure 3A), 15 metabolites were increased and two amino acids were decreased in HRP urine in comparison to INF pigs. Saliva displayed a somewhat opposite pattern, with 14 metabolites decreased and three amino acids increased in HRP pigs in comparison to INF pigs. Serum appeared to have a more variable pattern in comparison to saliva and urine, with 3 amino acids or derivatives increased and 6 decreased in HRP in comparison to INF.

Of the fatty-acid oxidation-related carnitine metabolites (Figure 4B), acetyl carnitine (C0) was significantly increased in HRP pigs across all three biofluids, and acetyl-L-carnitine (C2) was increased in HRP urine in comparison to the INF group. In urine, the significant short-chain derivatives (C3-C5) were all increased in HRP sows. Similarly, the majority of significant medium-chain (C6-C12) and long-chain (C14-C20) derivatives in were increased in HRP urine in comparison to INF urine. In saliva, the pattern was less clear with approximately equal short chain, medium chain and long chain derivatives increased and decreased in HRP sows incomparison to INF sows. In serum, the three significant short-chain derivatives were increased and one was decreased in HRP serum and the significant medium-chain derivative C6 and long-chain derivative C18:1 were decreased in HRP serum in comparison to INF.

Of the phospholipids, all glycerophospholipids (LYSOC) and phosphatidylcholines (PC) (Figure 4C) were present at higher concentrations in HRP pig serum in comparison to INF pigs. In saliva, all significant glycerophospholipids and phosphatidylcholines, besides PC38:6AA, were higher in HRP pigs in comparison to INF pigs. In urine, the majority (9) of glycerophospholipid and phosphatidylcholine were increased in HRP pigs, with the exception of PC38:6AA and PC30:0AA which were decreased in HRP pigs in comparison to INF pigs. Sphingomyelin (SM) phospholipids increased in HRP pig urine and decreased in HRP saliva compared to INF pigs. 

Of the steroid hormones (Figure 4D), no differences between HRP and INF pigs were observed in serum. Cortisol and testosterone were significantly increased and progesterone significantly decreased in both HRP urine and saliva. Androsterone, cortisone, 17-hydroxyprogesterone and dehydroepiandrosterone were higher in HRP urine in comparison to INF. Androstenedione and estrone were lower in HRP saliva in comparison to INF.

### 3.3. Biomarker Selection and Model Performance

**Figure 5:**
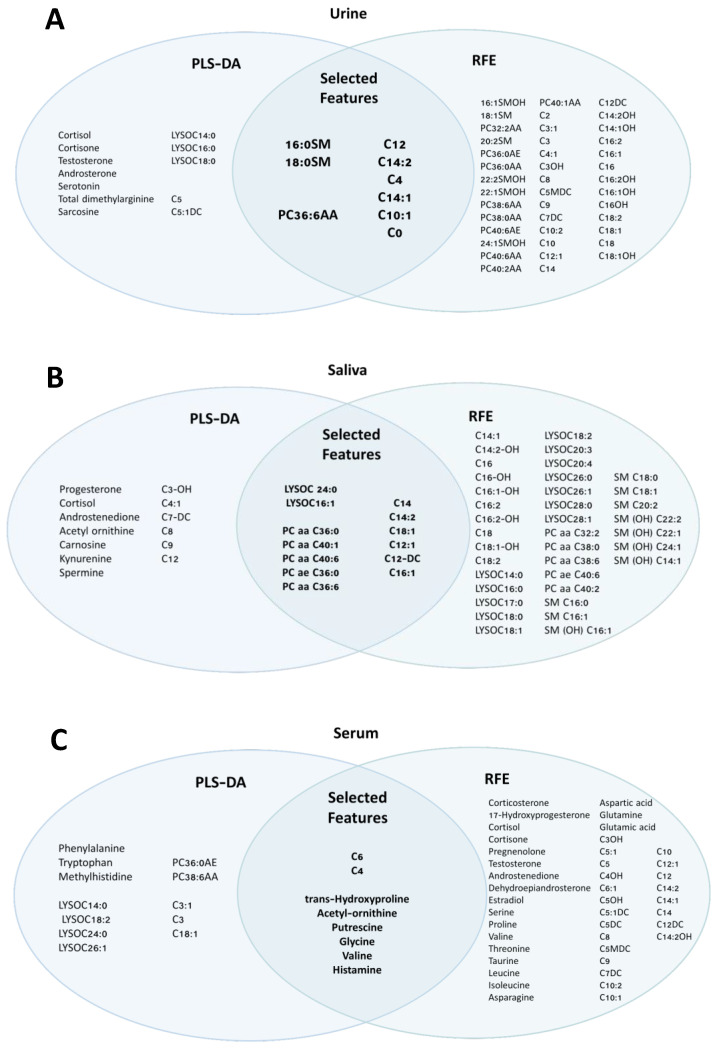
Selected features for ROC-AUC diagnostic classifier for urine (**A**), saliva (**B**), and serum (**C**). Features were selected via PLS-DA (VIP ≥ 1.25 + *p* < 0.05) and RFE.

**Figure 6:**
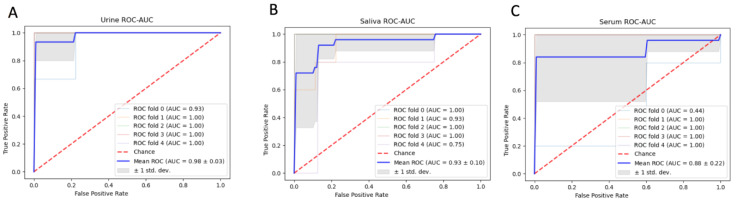
ROC-AUC diagnostic predictors using the selected biomarker candidates for urine (**A**), saliva (**B**) and serum (**C**). The urine model appeared to be the most diagnostic (AUC = 0.98), followed by saliva (AUC = 0.93) and serum (AUC = 0.88). The red dotted line depicts a classifier that has no predictive ability (AUC = 0.5) and the blue solid line depicts the average predictive ability of the diagnostic predictor over five stratified K-fold cross-validations.

Significant metabolites (*p* < 0.05) that had a VIP ≥ 1.25 from the PLS-DA models and were selected in RFE were chosen as potential biomarker candidates and used in the ROC curve modelling of each biofluid (Figure 5). The selected serum biomarkers appeared to favor metabolites related to amino acid and fatty acid oxidation, with six amino acids and two carnitines selected. Selected urine and saliva biomarkers appeared to favour metabolites related to membrane lipid metabolism and fatty acid oxidation, with urine including three phospholipids and six carnitines and saliva including seven phospholipids and six medium- and long-chain carnitine derivatives. Interestingly, the short-chain acyl carnitine derivative C4 was included in both urine and serum diagnostic models and the long-chain acyl carnitine derivative C14:2 was included in both urine and saliva diagnostic models. ROC-AUC models were used to evaluate the diagnostic ability of the potential biomarker candidates separating the metabolic profiles of HRP and INF reproductive groups. Urine had an average accuracy of 98% based on 100 cross-validations using the AUC as the performance measure (Figure 6A). Saliva and serum followed, with average accuracies of 93% and 88%, respectively (Figure 6B,C). 

## 4. Discussion

This study demonstrates that HRP and INF sows have diverging amino acid, fatty acid oxidation, and membrane lipid metabolic profiles in urine, saliva and serum. This supports that metabolism is widely implicated in sow fertility, and its changes can be clearly reflected in urine, saliva and serum biofluids. The urine and saliva PLS-DAs in this study have stronger metabolic separation of HRP and INF groups and higher replicability in comparison to the serum PLS-DA. This pattern is also reflected in the RFE selection of 50 features, with urine and saliva features out-preforming serum features. This is supported by urine and saliva’s superior accuracy in RFE, and superior accuracy and cross validation performance in PLS-DA, demonstrating high R2 performance measures and lower error measures in comparison to serum’s performance. 

The urine metabolite model appeared to be the most discriminatory followed by the serum and saliva metabolite models. Overall, it appears that all three biofluids could be used for predictive modelling of reproductive state between pigs to determine their reproductive potential, with urine having a slight advantage over saliva and serum. The panel of candidate biomarkers identified across the three biofluids examined in this study suggests that fatty acid oxidation metabolism, speficially carnitines, is involved in the cross-talk between metabolism and reproductive potential in sows and could be very useful in predicting the reproductive potential level of a female pig, specifically in saliva, urine and serum biofluids. Additionally, amino acid metabolism in serum and membrane lipid metabolism in saliva and urine had metabolites appear as candidate biomarkers, also suggesting that they could be useful for prediction purposes. Future research examining the predictive ability of these selected candidate biomarkers in an independent population of sows and gilts would need to be performed to further validate this finding and thereby classify these metabolites as diagnostic biomarkers of sow fertility.

Urine and saliva had the most significant metabolic changes between HRP and INF groups (69 and 66 significant metabolites, respectively) followed by serum (21 significant metabolites). It appeared that saliva had the most variable results across metabolite groups, whereas urine and serum had more distinct trends. The variable saliva metabolite patterns may be explained by the instability and variability in saliva metabolites of cellular origin and cellular debris [42,43]. However, saliva has an advantage over serum and urine it is more reflective of functional levels or “bioavailable” steroid hormones in the body [44,45]. A large proportion of steroid hormones are strongly bound by specific globulins in the blood but are inactive in this bound state. Saliva levels of steroid hormones are thought to reflect the unbound state, therefore better reflecting the bioavailable levels [44,45]. Urine is considered a metabolic waste product, accumulating over a prolonged period and is mainly free of interfering proteins and lipids, shedding light on the endogenous waste metabolites of an organism [14]. Serum is under greater homeostatic control than saliva and urine, making it more reflective of the metabolic condition as it subject to less external variability [18], and in the context of female reproduction, its metabolic content can reflect the content of follicular fluid, and thus, the metabolite concentration surrounding the oocytes [46,47]. However, there are disadvantages to using serum in the context of metabolomics and biomarker discovery; serum provides a “snapshot” of the individual at a specific time-point, inviting increased variability between individual samples based on time of sampling and providing more limited metabolic information. Taken together, due to the inherent properties of the sampled biofluids, the higher prevalence of urine and saliva significance but not serum may be attributed to accumulation of endogenous metabolic “waste” products in urine and the systemic reflection of bio-available hormones in saliva, magnifying and better reflecting even the slightest metabolic differences between the HRP and INF groups.

Taking the feature selection, diagnostic performance and nature of the biofluids together, urine and saliva may be better suited biofluid candidates for the diagnostic prediction of reproductive potential level in female pigs using metabolomics. Serum provides some diagnostic ability and could be used as a support to the urine and serum biomarkers, however further work needs to be done to improve the predictive ability of serum biomarkers.

### 4.1. Reduced Amino Acid Pool in INF Pigs

The sufficient uptake, metabolism and transport of amino acids is essential for proper mammalian embryo development, oocyte quality and uterine environment. Amino acids are highly prevalent in the reproductive tract, highlighting their importance [48,49]. The available endogenous pool of embryonic amino acids turns over every 72 h, and this turnover has been used as a measure of mammalian oocyte and embryo viability [50,51]. Additionally, many conditions of female infertility, including endometriosis [52], PCOS [25], and recurrent miscarriage [53], implicate aberrant amino acid metabolism. The decreased levels of amino acid metabolites present INF urine may suggest overall insufficient amino acid sources or less active amino acid metabolism in INF pigs, possibly contributing to poor oocyte quality, embryo quality or uterine environment, contributing to their infertile phenotype as outlined above.

Chen et al. [30] examined the metabolomics of oocyte quality in low reproductive potential and normal reproductive potential sow follicular fluid, urine and serum, reporting that amino acids involved in glycine/serine/threonine, tryptophan, and aspartic acid metabolism were aberrant in lower reproductive performers with poor oocyte quality. These results are consistent with the findings in the present study. Our analysis also identified altered concentrations of the derivatives of the pathways highlighted by Chen et al. [30], and these results are consistent with their physiological roles in female mammalian reproduction.

Many early mammalian reproductive failures are related to derangements of one-carbon metabolism (methylation), which modulates DNA methylation by controlling methyl availability [54,55]. Mammalian oocytes and embryos go through dynamic alterations in their DNA methylation for maturation and development [56]. In the present study, amino acid metabolites involved in the transport or excretion of methyl groups were increased in HRP urine and/or serum, including betaine, taurine, glycine and methionine (reviewed in [57]). Asymmetric and total dimethyl arginine are also increased in HRP urine; this excretion is important to activate endothelial nitric oxide synthase [58] allowing for regulation of vascularization in embryo development and implantation [59,60,61]. These results indicate that HRP pigs could have higher levels of methyl group excretion in comparison to INF pigs. Due to the importance of the modulation of DNA methylation in oocyte and embryo development, aberrant DNA methylation in INF pigs could lead to impaired oocyte maturation and embryo development. Further research should aim to explore the DNA methylation state of oocytes or embryos from infertile and highly reproducing pigs to explore how one-carbon metabolism and methylation could result in the studied phenotypes.

### 4.2. L-Carnitine and Acetyl-L-Carnitine Increased for Fatty Acid Oxidation and ROS Protection in HRP Pigs

L-carnitine (C0) and acetyl-L-carnitine (C2) play an essential physiological role in the transportation of long-chain fatty acids from the cytosol to the mitochondria where they are oxidized for energy production [62]. Fatty acid oxidation by the cumulus oocyte complex (COC) is vital for oocyte and embryo maturation, quality and competence (reviewed in [63]) as well as early embryo development, making L-carnitine and acetyl-L-carnitine essential cofactors for competent development [64]. Porcine oocytes, in comparison to other domestic animals, such as cattle and sheep, are extremely lipid rich [65] and rely heavily on fatty acid oxidation for developmental competence [66]. During the in vitro maturation of porcine oocytes, a clear benefit of L-carnitine has been highlighted in numerous studies [67,68,69,70], with its supplementation significantly increasing oocyte mitochondrial activity [68] (reviewed in [71]). In addition to these beneficial roles, L-carnitine (C0) and acetyl-L-carnitine (C2) work as antioxidants to regulate and protect the metabolic status of the female reproductive tract and associated tissues, neutralizing and ridding of the damaging free radicals and damaging intermediary molecules [5,72], which are implicated in female mammalian infertility [73,74]. In practice, antioxidant application to gestating sows can increase litter size [75], and L-carnitine was found to reduce ROS levels in porcine oocytes during in vitro maturation [70], highlighting the importance of the regulation of ROS in infertility. The increase of C0 and C2 in HRP sow biofluids in the present study may reflect an increase of fatty acid oxidation capacity and ROS protection. This state would promote HRP oocyte development and embryo viability, leading to better reproductive outcomes. Additioally, in fatty acid oxidation, L-carnitine and acetyl-L-carnitine are converted to short-chain (C3-C5) medium chain (C6-C12) and long-chain (C14-C20) acylcarnitine derivatives [62]. The increased levels of short chain acylcarnitine derivatives (C3-C5) found in HRP urine and serum and altered levels of medium- (C6-C12) and long-chain (C14-C20) derivatives in saliva urine and serum support the notion that HRP sows better exploit carnitines for fatty acid oxidation in comparison to INF sows. 

### 4.3. Disrupted Membrane Lipid Metabolism in INF Pigs

Phospholipids contribute significantly to mammalian cellular membranes, controlling diffusion and transportation of molecules in and out of the cell, regulation of signal transduction, cell communication, and complex functioning [76,77]. The permeability and quality of the oocyte’s plasma membrane contributes significantly to early development and fertilization potential, and have been suggested as determinants of successful fertilization of oocytes in vitro [78]. Additionally, positive pregnancy outcomes have been associated with active membrane lipid metabolism with an overall decrease in triacylglycerol levels and a shift to increases in all membrane lipids, including glycosphingolipids, lysophospholipids, and sphingomyelins in follicular fluid [79]. Consistent with this notion, the majority of significant phospholipids were present at a higher concentration in HRP sow saliva, urine and serum in comparison to INF sows. Interestingly, four of these discriminant lipids, SM16:0, LYSOC18:0, LYSOC18:1, and LYSOC18:2, have been previously identified as possible predictors for successful pregnancy in follicular fluid, [79]. It is possible that the INF sows, due to disruption of overall phospholipid synthesis and/or membrane lipid metabolism, have poor quality oocytes with increased permeability, lending to unsuccessful fertilization in vivo. Accordingly, membrane lipid metabolism is highly disturbed in unexplained infertility with increased triacylglycerol levels, decreased phospholipids and sphingomyelin in follicular fluid [80], and in PCOS with decreased glycerophospholipids and sphingomyelin in follicular fluid [81], decreased lysophosphatidylcholines in serum and increased lysophosphatidylcholines in urine samples [82]. Overall, the significant differences in phospholipid concentrations between HRP and INF sow biofluids imply their involvement in female mammalian fertility. In the future, triacylglycerides, precursors to membrane lipids, should be examined to get a more accurate overview of the state of membrane lipid metabolism.

### 4.4. Beneficial Levels of Steroid Hormones HRP Pigs

Androgens and estrogens are important in the regulation of the mechanisms involved in oocyte maturation and ovulation in follicle development and maintenance of embryo quality (reviewed in [83]). Free androgen index has been found to positively correlate with ovarian follicle count [84], serum testosterone levels have been found to positively correlate with number of oocytes and available embryos [85,86], and embryo implantation rate [87], suggesting that lower levels of testosterone and androgens are associated with poor oocyte and embryo quality. Accordingly in pigs, the number of corpus lutea per follicle and ovulation rate of gilts increased after testosterone injection [88,89]. The positive roles of testosterone in female mammalian fertility supports the idea that HRP pigs are in better reproductive condition than the INF pigs due to relatively higher androgen levels in the form of testosterone, androsterone and DHEA. Estrogens play a role in the development and maturation of oocytes, corpus luteum formation, embryo development and implantation [90,91]. In the present study, estradiol concentrations were not measured in saliva and urine, but reduced levels of estrone were found in HRP pig saliva. It is suggested that the decreased concentration of estrone in HRP saliva is due to increased conversion to estradiol via the enzyme 17 beta-hydroxysteroid dehydrogenase (HSD-17β) [92]. Increased estradiol may contribute to the healthy fertile HRP phenotype expected. Future research should measure the levels of estradiol in HRP and INF phenotypes to support this speculation.

Interestingly, higher levels of glucocorticoids were found in HRP in comparison to INF pigs saliva and urine. Even though it has been reported that high levels of glucocorticoids are associated with negative reproductive outcomes related to oocytes [93,94,95], there is evidence supporting neutral or positive roles of these hormones in female reproduction. For example, the exposure of mouse oocytes directly to physiological or stress-induced concentrations of cortisol [94,96] or corticosterone [97] during in vitro maturation had no negative effect on nuclear maturation and embryo development and no evidence was found to support disruption of ovulation, conception, or number of embryos in gilts by stress or cortisol injection during estrus [98]. Interestingly, a 2-fold higher concentration of cortisol in the follicular fluid of follicles containing mature oocytes compared to immature oocyte containing follicles in an IVF trial, suggesting a positive role in maturation of oocytes [99]. Overall, it remains to be determined exactly how glucocorticoids effect oocyte and embryo competence in mammals. Neverless, the absence of increased glucocorticoid detected in serum suggest that their direct influence to the oocyte, and consequently fertility, is minimal.

## 5. Conclusions

Our study has characterized the urinary, salivary and serum metabolome simultaneously and identified potential candidate biomarkers in high reproductive potential and infertile sows. We report that amino acid, fatty acid oxidation, membrane lipid components, and steroid hormones were found to be different between HRP and INF sow biofluids. For biomarker identification purposes, the present study supports that urine may be a superior biofluid over saliva and serum for accurate reflection of the female pig’s metabolomic state and prediction of fertility potential, supported by the univariate, PLS-DA, RFE, and ROC-AUC analyses. The candidate biomarkers of sow reproductive potential identified in this study suggest that future studies should focus on fatty acid oxidation related metabolites in all three biofluids, and amino acid metabolism in serum, and membrane lipids in urine and saliva. Our results may also provide insight into characterizing metabolic phenotypes that are favourable or unfavourable for successful reproduction or fertility in other mammalian species, including humans. It also marks the first study completed with a large sample size of less-invasive biofluids, in comparison to previous termination studies focusing on follicular fluid and ovarian tissue. This work lays the foundation for improving existing gilt and sow selection protocols, and thus more profitable production in the pork industry. For real-world application and diagnostic purposes, further studies should focus on testing the candidate biomarkers identified in the present study in a novel population of pig samples to measure diagnostic ability.

## Data Availability

The data presented in this study are available on request from the corresponding author. Data is not publicly available due to its proprietary nature.

## Supplementary Materials

The following supporting information can be downloaded at: https://www.mdpi.com/article/10.3390/metabo12111045/s1, Table S1: Panel of serum metabolites analyzed after data pre-processing steps (125 metabolites total). Table S2: Panel of urine metabolites analyzed after data pre-processing steps (123 metabolites total). Table S3: Panel of saliva metabolites analyzed after data pre-processing steps (155 metabolites total). Table S4: Urine metabolites found at significantly different (*p* < 0.05) concentrations between HRP and INF sows. Table S5: Saliva metabolites found at significantly different (*p* < 0.05) concentrations between HRP and INF sows. Table S6: Serum metabolites found at significantly different (*p* < 0.05) concentrations between HRP and INF sows.
