# Supplementary material for: Identification of Candidate Salivary, Urinary and Serum Metabolic Biomarkers for High Litter Size Potential in Sows (Sus scrofa)
Source: Metabolites. 2022 Oct 30;12(11):1045. doi: 10.3390/metabo12111045 (PMC9697495; doi:10.3390/metabo12111045)
Supplement: Supplementary file 1 [file metabolites-12-01045-s001.zip › SuppementaryTables.pptx]

## Slide 1
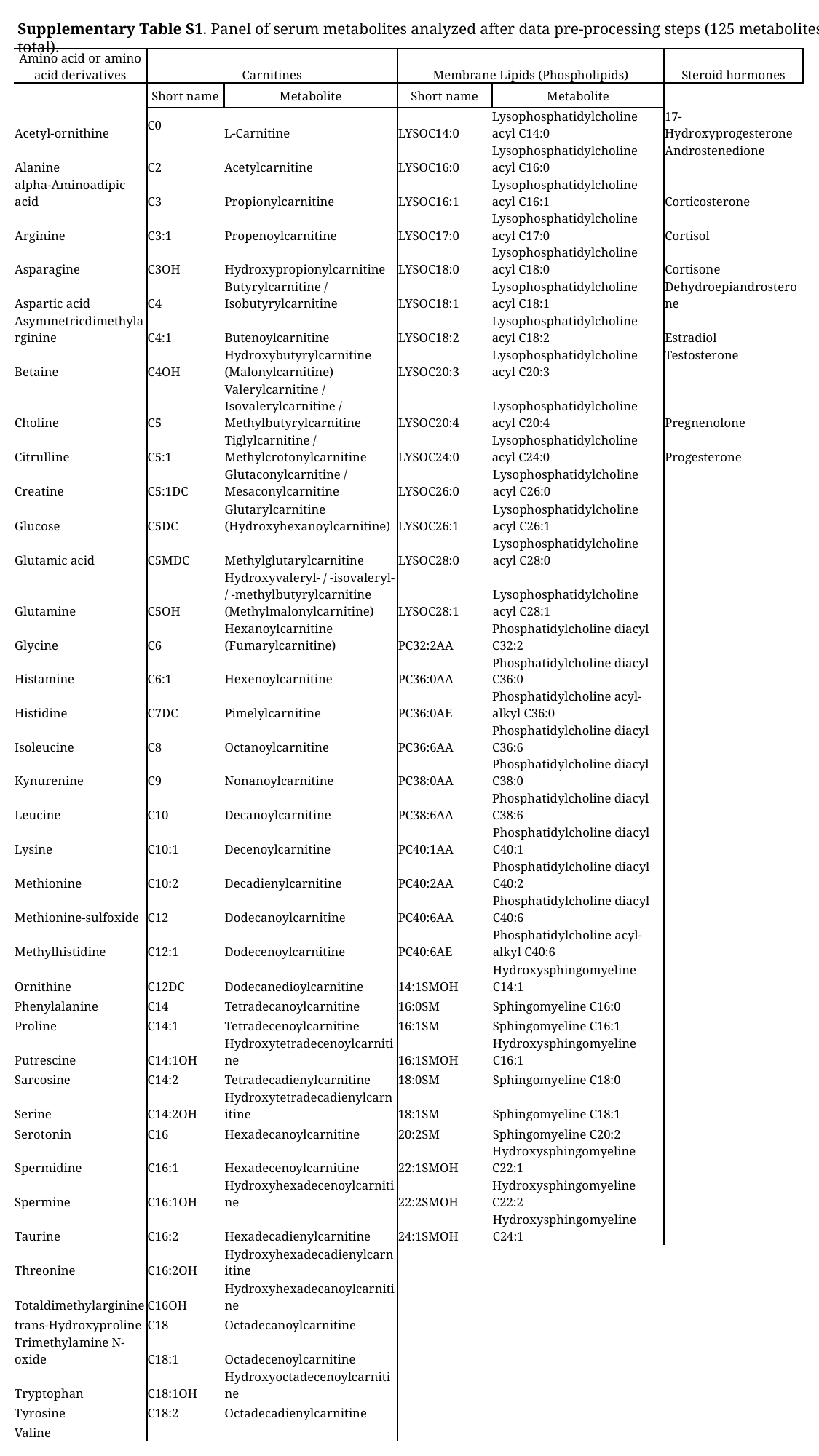

Supplementary Table S1. Panel of serum metabolites analyzed after data pre-processing steps (125 metabolites total).
| Amino acid or amino acid derivatives | Carnitines | | Membrane Lipids (Phospholipids) | | Steroid hormones |
| --- | --- | --- | --- | --- | --- |
| Acetyl-ornithine | Short name | Metabolite | Short name | Metabolite | 17-Hydroxyprogesterone |
| | C0 | L-Carnitine | LYSOC14:0 | Lysophosphatidylcholine acyl C14:0 | |
| Alanine | C2 | Acetylcarnitine | LYSOC16:0 | Lysophosphatidylcholine acyl C16:0 | Androstenedione |
| alpha-Aminoadipic acid | C3 | Propionylcarnitine | LYSOC16:1 | Lysophosphatidylcholine acyl C16:1 | Corticosterone |
| Arginine | C3:1 | Propenoylcarnitine | LYSOC17:0 | Lysophosphatidylcholine acyl C17:0 | Cortisol |
| Asparagine | C3OH | Hydroxypropionylcarnitine | LYSOC18:0 | Lysophosphatidylcholine acyl C18:0 | Cortisone |
| Aspartic acid | C4 | Butyrylcarnitine / Isobutyrylcarnitine | LYSOC18:1 | Lysophosphatidylcholine acyl C18:1 | Dehydroepiandrosterone |
| Asymmetricdimethylarginine | C4:1 | Butenoylcarnitine | LYSOC18:2 | Lysophosphatidylcholine acyl C18:2 | Estradiol |
| Betaine | C4OH | Hydroxybutyrylcarnitine (Malonylcarnitine) | LYSOC20:3 | Lysophosphatidylcholine acyl C20:3 | Testosterone |
| Choline | C5 | Valerylcarnitine / Isovalerylcarnitine / Methylbutyrylcarnitine | LYSOC20:4 | Lysophosphatidylcholine acyl C20:4 | Pregnenolone |
| Citrulline | C5:1 | Tiglylcarnitine / Methylcrotonylcarnitine | LYSOC24:0 | Lysophosphatidylcholine acyl C24:0 | Progesterone |
| Creatine | C5:1DC | Glutaconylcarnitine / Mesaconylcarnitine | LYSOC26:0 | Lysophosphatidylcholine acyl C26:0 | |
| Glucose | C5DC | Glutarylcarnitine (Hydroxyhexanoylcarnitine) | LYSOC26:1 | Lysophosphatidylcholine acyl C26:1 | |
| Glutamic acid | C5MDC | Methylglutarylcarnitine | LYSOC28:0 | Lysophosphatidylcholine acyl C28:0 | |
| Glutamine | C5OH | Hydroxyvaleryl- / -isovaleryl- / -methylbutyrylcarnitine (Methylmalonylcarnitine) | LYSOC28:1 | Lysophosphatidylcholine acyl C28:1 | |
| Glycine | C6 | Hexanoylcarnitine (Fumarylcarnitine) | PC32:2AA | Phosphatidylcholine diacyl C32:2 | |
| Histamine | C6:1 | Hexenoylcarnitine | PC36:0AA | Phosphatidylcholine diacyl C36:0 | |
| Histidine | C7DC | Pimelylcarnitine | PC36:0AE | Phosphatidylcholine acyl-alkyl C36:0 | |
| Isoleucine | C8 | Octanoylcarnitine | PC36:6AA | Phosphatidylcholine diacyl C36:6 | |
| Kynurenine | C9 | Nonanoylcarnitine | PC38:0AA | Phosphatidylcholine diacyl C38:0 | |
| Leucine | C10 | Decanoylcarnitine | PC38:6AA | Phosphatidylcholine diacyl C38:6 | |
| Lysine | C10:1 | Decenoylcarnitine | PC40:1AA | Phosphatidylcholine diacyl C40:1 | |
| Methionine | C10:2 | Decadienylcarnitine | PC40:2AA | Phosphatidylcholine diacyl C40:2 | |
| Methionine-sulfoxide | C12 | Dodecanoylcarnitine | PC40:6AA | Phosphatidylcholine diacyl C40:6 | |
| Methylhistidine | C12:1 | Dodecenoylcarnitine | PC40:6AE | Phosphatidylcholine acyl-alkyl C40:6 | |
| Ornithine | C12DC | Dodecanedioylcarnitine | 14:1SMOH | Hydroxysphingomyeline C14:1 | |
| Phenylalanine | C14 | Tetradecanoylcarnitine | 16:0SM | Sphingomyeline C16:0 | |
| Proline | C14:1 | Tetradecenoylcarnitine | 16:1SM | Sphingomyeline C16:1 | |
| Putrescine | C14:1OH | Hydroxytetradecenoylcarnitine | 16:1SMOH | Hydroxysphingomyeline C16:1 | |
| Sarcosine | C14:2 | Tetradecadienylcarnitine | 18:0SM | Sphingomyeline C18:0 | |
| Serine | C14:2OH | Hydroxytetradecadienylcarnitine | 18:1SM | Sphingomyeline C18:1 | |
| Serotonin | C16 | Hexadecanoylcarnitine | 20:2SM | Sphingomyeline C20:2 | |
| Spermidine | C16:1 | Hexadecenoylcarnitine | 22:1SMOH | Hydroxysphingomyeline C22:1 | |
| Spermine | C16:1OH | Hydroxyhexadecenoylcarnitine | 22:2SMOH | Hydroxysphingomyeline C22:2 | |
| Taurine | C16:2 | Hexadecadienylcarnitine | 24:1SMOH | Hydroxysphingomyeline C24:1 | |
| Threonine | C16:2OH | Hydroxyhexadecadienylcarnitine | | | |
| Totaldimethylarginine | C16OH | Hydroxyhexadecanoylcarnitine | | | |
| trans-Hydroxyproline | C18 | Octadecanoylcarnitine | | | |
| Trimethylamine N-oxide | C18:1 | Octadecenoylcarnitine | | | |
| Tryptophan | C18:1OH | Hydroxyoctadecenoylcarnitine | | | |
| Tyrosine | C18:2 | Octadecadienylcarnitine | | | |
| Valine | | | | | |

## Slide 2
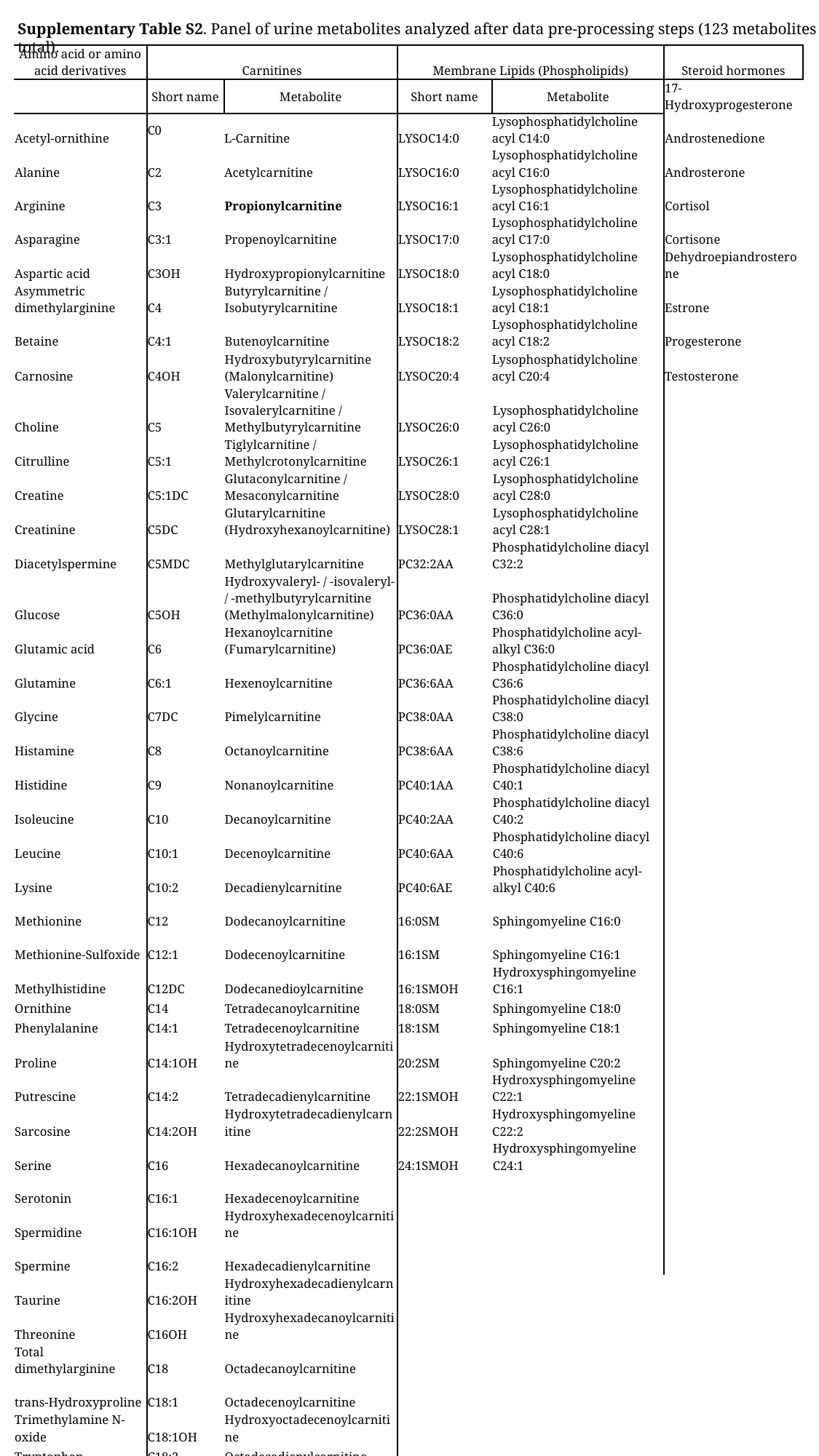

Supplementary Table S2. Panel of urine metabolites analyzed after data pre-processing steps (123 metabolites total).
| Amino acid or amino acid derivatives | Carnitines | | Membrane Lipids (Phospholipids) | | Steroid hormones |
| --- | --- | --- | --- | --- | --- |
| | Short name | Metabolite | Short name | Metabolite | 17-Hydroxyprogesterone |
| Acetyl-ornithine | C0 | L-Carnitine | LYSOC14:0 | Lysophosphatidylcholine acyl C14:0 | Androstenedione |
| Alanine | C2 | Acetylcarnitine | LYSOC16:0 | Lysophosphatidylcholine acyl C16:0 | Androsterone |
| Arginine | C3 | Propionylcarnitine | LYSOC16:1 | Lysophosphatidylcholine acyl C16:1 | Cortisol |
| Asparagine | C3:1 | Propenoylcarnitine | LYSOC17:0 | Lysophosphatidylcholine acyl C17:0 | Cortisone |
| Aspartic acid | C3OH | Hydroxypropionylcarnitine | LYSOC18:0 | Lysophosphatidylcholine acyl C18:0 | Dehydroepiandrosterone |
| Asymmetric dimethylarginine | C4 | Butyrylcarnitine / Isobutyrylcarnitine | LYSOC18:1 | Lysophosphatidylcholine acyl C18:1 | Estrone |
| Betaine | C4:1 | Butenoylcarnitine | LYSOC18:2 | Lysophosphatidylcholine acyl C18:2 | Progesterone |
| Carnosine | C4OH | Hydroxybutyrylcarnitine (Malonylcarnitine) | LYSOC20:4 | Lysophosphatidylcholine acyl C20:4 | Testosterone |
| Choline | C5 | Valerylcarnitine / Isovalerylcarnitine / Methylbutyrylcarnitine | LYSOC26:0 | Lysophosphatidylcholine acyl C26:0 | |
| Citrulline | C5:1 | Tiglylcarnitine / Methylcrotonylcarnitine | LYSOC26:1 | Lysophosphatidylcholine acyl C26:1 | |
| Creatine | C5:1DC | Glutaconylcarnitine / Mesaconylcarnitine | LYSOC28:0 | Lysophosphatidylcholine acyl C28:0 | |
| Creatinine | C5DC | Glutarylcarnitine (Hydroxyhexanoylcarnitine) | LYSOC28:1 | Lysophosphatidylcholine acyl C28:1 | |
| Diacetylspermine | C5MDC | Methylglutarylcarnitine | PC32:2AA | Phosphatidylcholine diacyl C32:2 | |
| Glucose | C5OH | Hydroxyvaleryl- / -isovaleryl- / -methylbutyrylcarnitine (Methylmalonylcarnitine) | PC36:0AA | Phosphatidylcholine diacyl C36:0 | |
| Glutamic acid | C6 | Hexanoylcarnitine (Fumarylcarnitine) | PC36:0AE | Phosphatidylcholine acyl-alkyl C36:0 | |
| Glutamine | C6:1 | Hexenoylcarnitine | PC36:6AA | Phosphatidylcholine diacyl C36:6 | |
| Glycine | C7DC | Pimelylcarnitine | PC38:0AA | Phosphatidylcholine diacyl C38:0 | |
| Histamine | C8 | Octanoylcarnitine | PC38:6AA | Phosphatidylcholine diacyl C38:6 | |
| Histidine | C9 | Nonanoylcarnitine | PC40:1AA | Phosphatidylcholine diacyl C40:1 | |
| Isoleucine | C10 | Decanoylcarnitine | PC40:2AA | Phosphatidylcholine diacyl C40:2 | |
| Leucine | C10:1 | Decenoylcarnitine | PC40:6AA | Phosphatidylcholine diacyl C40:6 | |
| Lysine | C10:2 | Decadienylcarnitine | PC40:6AE | Phosphatidylcholine acyl-alkyl C40:6 | |
| Methionine | C12 | Dodecanoylcarnitine | 16:0SM | Sphingomyeline C16:0 | |
| Methionine-Sulfoxide | C12:1 | Dodecenoylcarnitine | 16:1SM | Sphingomyeline C16:1 | |
| Methylhistidine | C12DC | Dodecanedioylcarnitine | 16:1SMOH | Hydroxysphingomyeline C16:1 | |
| Ornithine | C14 | Tetradecanoylcarnitine | 18:0SM | Sphingomyeline C18:0 | |
| Phenylalanine | C14:1 | Tetradecenoylcarnitine | 18:1SM | Sphingomyeline C18:1 | |
| Proline | C14:1OH | Hydroxytetradecenoylcarnitine | 20:2SM | Sphingomyeline C20:2 | |
| Putrescine | C14:2 | Tetradecadienylcarnitine | 22:1SMOH | Hydroxysphingomyeline C22:1 | |
| Sarcosine | C14:2OH | Hydroxytetradecadienylcarnitine | 22:2SMOH | Hydroxysphingomyeline C22:2 | |
| Serine | C16 | Hexadecanoylcarnitine | 24:1SMOH | Hydroxysphingomyeline C24:1 | |
| Serotonin | C16:1 | Hexadecenoylcarnitine | | | |
| Spermidine | C16:1OH | Hydroxyhexadecenoylcarnitine | | | |
| Spermine | C16:2 | Hexadecadienylcarnitine | | | |
| Taurine | C16:2OH | Hydroxyhexadecadienylcarnitine | | | |
| Threonine | C16OH | Hydroxyhexadecanoylcarnitine | | | |
| Total dimethylarginine | C18 | Octadecanoylcarnitine | | | |
| trans-Hydroxyproline | C18:1 | Octadecenoylcarnitine | | | |
| Trimethylamine N-oxide | C18:1OH | Hydroxyoctadecenoylcarnitine | | | |
| Tryptophan | C18:2 | Octadecadienylcarnitine | | | |
| Tyramine | | | | | |
| Tyrosine | | | | | |
| Valine | | | | | |

## Slide 3
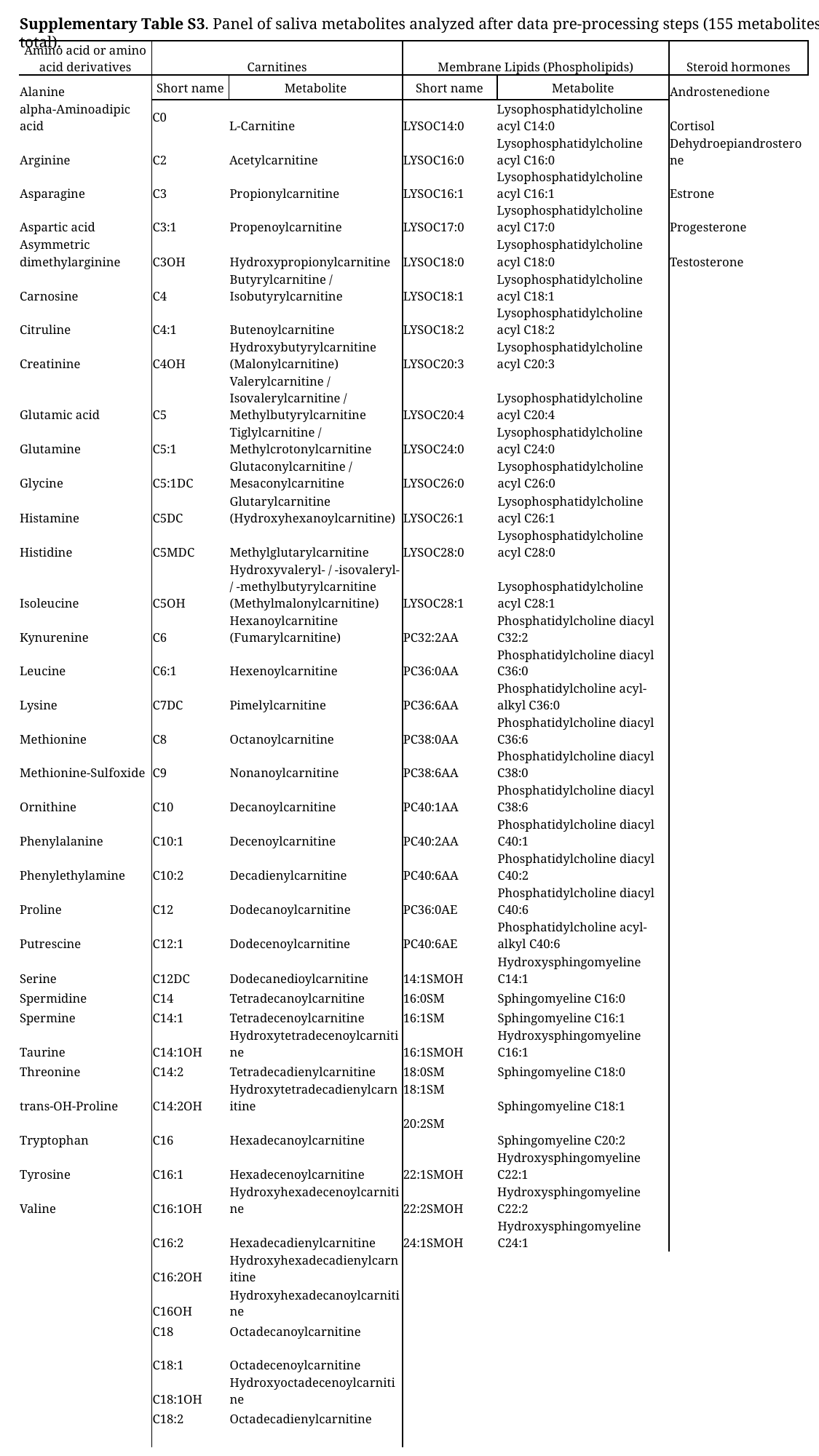

Supplementary Table S3. Panel of saliva metabolites analyzed after data pre-processing steps (155 metabolites total).
| Amino acid or amino acid derivatives | Carnitines | | Membrane Lipids (Phospholipids) | | Steroid hormones |
| --- | --- | --- | --- | --- | --- |
| Alanine | Short name | Metabolite | Short name | Metabolite | Androstenedione |
| alpha-Aminoadipic acid | C0 | L-Carnitine | LYSOC14:0 | Lysophosphatidylcholine acyl C14:0 | Cortisol |
| Arginine | C2 | Acetylcarnitine | LYSOC16:0 | Lysophosphatidylcholine acyl C16:0 | Dehydroepiandrosterone |
| Asparagine | C3 | Propionylcarnitine | LYSOC16:1 | Lysophosphatidylcholine acyl C16:1 | Estrone |
| Aspartic acid | C3:1 | Propenoylcarnitine | LYSOC17:0 | Lysophosphatidylcholine acyl C17:0 | Progesterone |
| Asymmetric dimethylarginine | C3OH | Hydroxypropionylcarnitine | LYSOC18:0 | Lysophosphatidylcholine acyl C18:0 | Testosterone |
| Carnosine | C4 | Butyrylcarnitine / Isobutyrylcarnitine | LYSOC18:1 | Lysophosphatidylcholine acyl C18:1 | |
| Citruline | C4:1 | Butenoylcarnitine | LYSOC18:2 | Lysophosphatidylcholine acyl C18:2 | |
| Creatinine | C4OH | Hydroxybutyrylcarnitine (Malonylcarnitine) | LYSOC20:3 | Lysophosphatidylcholine acyl C20:3 | |
| Glutamic acid | C5 | Valerylcarnitine / Isovalerylcarnitine / Methylbutyrylcarnitine | LYSOC20:4 | Lysophosphatidylcholine acyl C20:4 | |
| Glutamine | C5:1 | Tiglylcarnitine / Methylcrotonylcarnitine | LYSOC24:0 | Lysophosphatidylcholine acyl C24:0 | |
| Glycine | C5:1DC | Glutaconylcarnitine / Mesaconylcarnitine | LYSOC26:0 | Lysophosphatidylcholine acyl C26:0 | |
| Histamine | C5DC | Glutarylcarnitine (Hydroxyhexanoylcarnitine) | LYSOC26:1 | Lysophosphatidylcholine acyl C26:1 | |
| Histidine | C5MDC | Methylglutarylcarnitine | LYSOC28:0 | Lysophosphatidylcholine acyl C28:0 | |
| Isoleucine | C5OH | Hydroxyvaleryl- / -isovaleryl- / -methylbutyrylcarnitine (Methylmalonylcarnitine) | LYSOC28:1 | Lysophosphatidylcholine acyl C28:1 | |
| Kynurenine | C6 | Hexanoylcarnitine (Fumarylcarnitine) | PC32:2AA | Phosphatidylcholine diacyl C32:2 | |
| Leucine | C6:1 | Hexenoylcarnitine | PC36:0AA | Phosphatidylcholine diacyl C36:0 | |
| Lysine | C7DC | Pimelylcarnitine | PC36:6AA | Phosphatidylcholine acyl-alkyl C36:0 | |
| Methionine | C8 | Octanoylcarnitine | PC38:0AA | Phosphatidylcholine diacyl C36:6 | |
| Methionine-Sulfoxide | C9 | Nonanoylcarnitine | PC38:6AA | Phosphatidylcholine diacyl C38:0 | |
| Ornithine | C10 | Decanoylcarnitine | PC40:1AA | Phosphatidylcholine diacyl C38:6 | |
| Phenylalanine | C10:1 | Decenoylcarnitine | PC40:2AA | Phosphatidylcholine diacyl C40:1 | |
| Phenylethylamine | C10:2 | Decadienylcarnitine | PC40:6AA | Phosphatidylcholine diacyl C40:2 | |
| Proline | C12 | Dodecanoylcarnitine | PC36:0AE | Phosphatidylcholine diacyl C40:6 | |
| Putrescine | C12:1 | Dodecenoylcarnitine | PC40:6AE | Phosphatidylcholine acyl-alkyl C40:6 | |
| Serine | C12DC | Dodecanedioylcarnitine | 14:1SMOH | Hydroxysphingomyeline C14:1 | |
| Spermidine | C14 | Tetradecanoylcarnitine | 16:0SM | Sphingomyeline C16:0 | |
| Spermine | C14:1 | Tetradecenoylcarnitine | 16:1SM | Sphingomyeline C16:1 | |
| Taurine | C14:1OH | Hydroxytetradecenoylcarnitine | 16:1SMOH | Hydroxysphingomyeline C16:1 | |
| Threonine | C14:2 | Tetradecadienylcarnitine | 18:0SM | Sphingomyeline C18:0 | |
| trans-OH-Proline | C14:2OH | Hydroxytetradecadienylcarnitine | 18:1SM | Sphingomyeline C18:1 | |
| Tryptophan | C16 | Hexadecanoylcarnitine | 20:2SM | Sphingomyeline C20:2 | |
| Tyrosine | C16:1 | Hexadecenoylcarnitine | 22:1SMOH | Hydroxysphingomyeline C22:1 | |
| Valine | C16:1OH | Hydroxyhexadecenoylcarnitine | 22:2SMOH | Hydroxysphingomyeline C22:2 | |
| | C16:2 | Hexadecadienylcarnitine | 24:1SMOH | Hydroxysphingomyeline C24:1 | |
| | C16:2OH | Hydroxyhexadecadienylcarnitine | | | |
| | C16OH | Hydroxyhexadecanoylcarnitine | | | |
| | C18 | Octadecanoylcarnitine | | | |
| | C18:1 | Octadecenoylcarnitine | | | |
| | C18:1OH | Hydroxyoctadecenoylcarnitine | | | |
| | C18:2 | Octadecadienylcarnitine | | | |
| | | | | | |

## Slide 4
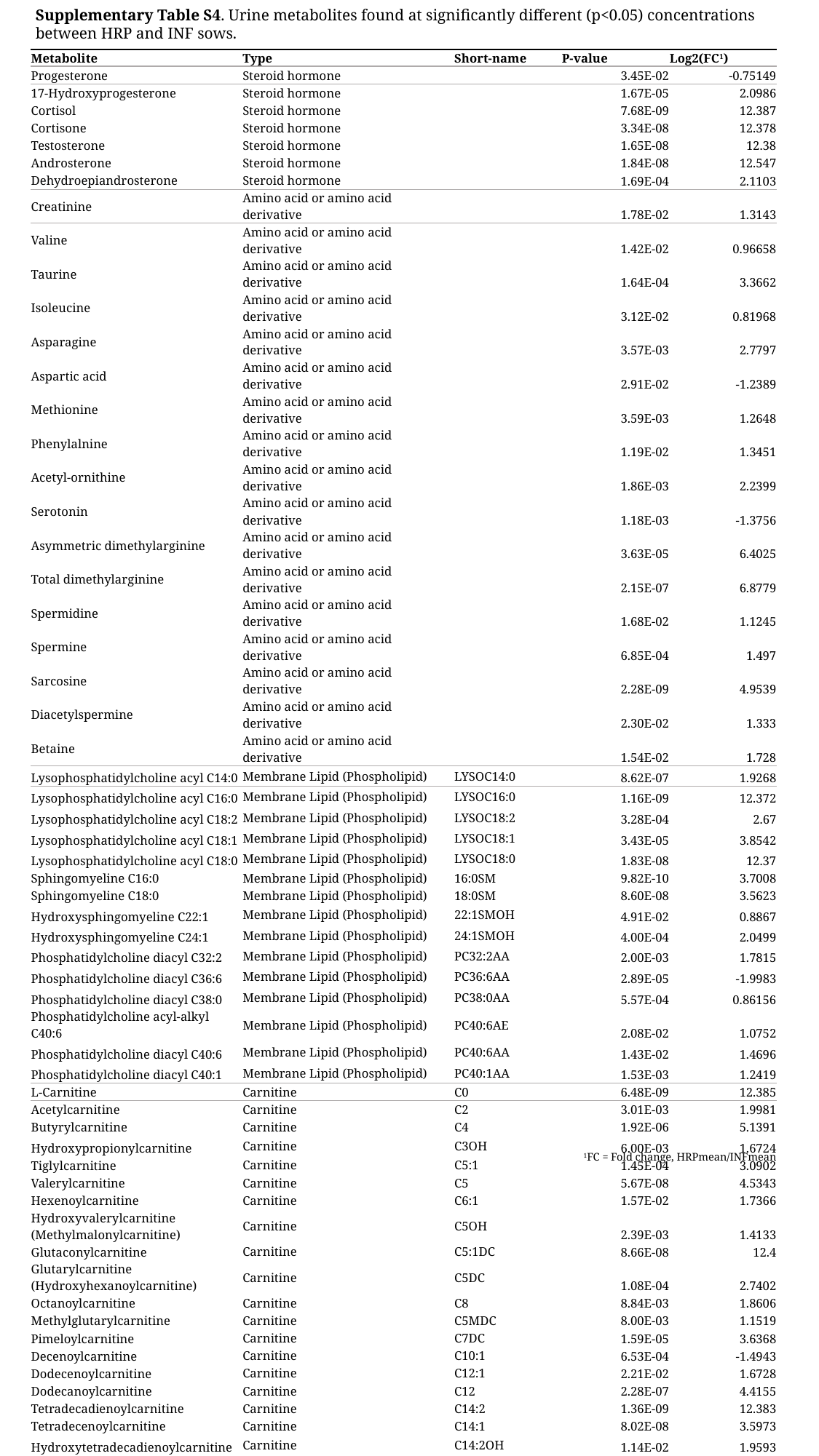

Supplementary Table S4. Urine metabolites found at significantly different (p<0.05) concentrations between HRP and INF sows.
| Metabolite | Type | Short-name | P-value | Log2(FC1) |
| --- | --- | --- | --- | --- |
| Progesterone | Steroid hormone | | 3.45E-02 | -0.75149 |
| 17-Hydroxyprogesterone | Steroid hormone | | 1.67E-05 | 2.0986 |
| Cortisol | Steroid hormone | | 7.68E-09 | 12.387 |
| Cortisone | Steroid hormone | | 3.34E-08 | 12.378 |
| Testosterone | Steroid hormone | | 1.65E-08 | 12.38 |
| Androsterone | Steroid hormone | | 1.84E-08 | 12.547 |
| Dehydroepiandrosterone | Steroid hormone | | 1.69E-04 | 2.1103 |
| Creatinine | Amino acid or amino acid derivative | | 1.78E-02 | 1.3143 |
| Valine | Amino acid or amino acid derivative | | 1.42E-02 | 0.96658 |
| Taurine | Amino acid or amino acid derivative | | 1.64E-04 | 3.3662 |
| Isoleucine | Amino acid or amino acid derivative | | 3.12E-02 | 0.81968 |
| Asparagine | Amino acid or amino acid derivative | | 3.57E-03 | 2.7797 |
| Aspartic acid | Amino acid or amino acid derivative | | 2.91E-02 | -1.2389 |
| Methionine | Amino acid or amino acid derivative | | 3.59E-03 | 1.2648 |
| Phenylalnine | Amino acid or amino acid derivative | | 1.19E-02 | 1.3451 |
| Acetyl-ornithine | Amino acid or amino acid derivative | | 1.86E-03 | 2.2399 |
| Serotonin | Amino acid or amino acid derivative | | 1.18E-03 | -1.3756 |
| Asymmetric dimethylarginine | Amino acid or amino acid derivative | | 3.63E-05 | 6.4025 |
| Total dimethylarginine | Amino acid or amino acid derivative | | 2.15E-07 | 6.8779 |
| Spermidine | Amino acid or amino acid derivative | | 1.68E-02 | 1.1245 |
| Spermine | Amino acid or amino acid derivative | | 6.85E-04 | 1.497 |
| Sarcosine | Amino acid or amino acid derivative | | 2.28E-09 | 4.9539 |
| Diacetylspermine | Amino acid or amino acid derivative | | 2.30E-02 | 1.333 |
| Betaine | Amino acid or amino acid derivative | | 1.54E-02 | 1.728 |
| Lysophosphatidylcholine acyl C14:0 | Membrane Lipid (Phospholipid) | LYSOC14:0 | 8.62E-07 | 1.9268 |
| Lysophosphatidylcholine acyl C16:0 | Membrane Lipid (Phospholipid) | LYSOC16:0 | 1.16E-09 | 12.372 |
| Lysophosphatidylcholine acyl C18:2 | Membrane Lipid (Phospholipid) | LYSOC18:2 | 3.28E-04 | 2.67 |
| Lysophosphatidylcholine acyl C18:1 | Membrane Lipid (Phospholipid) | LYSOC18:1 | 3.43E-05 | 3.8542 |
| Lysophosphatidylcholine acyl C18:0 | Membrane Lipid (Phospholipid) | LYSOC18:0 | 1.83E-08 | 12.37 |
| Sphingomyeline C16:0 | Membrane Lipid (Phospholipid) | 16:0SM | 9.82E-10 | 3.7008 |
| Sphingomyeline C18:0 | Membrane Lipid (Phospholipid) | 18:0SM | 8.60E-08 | 3.5623 |
| Hydroxysphingomyeline C22:1 | Membrane Lipid (Phospholipid) | 22:1SMOH | 4.91E-02 | 0.8867 |
| Hydroxysphingomyeline C24:1 | Membrane Lipid (Phospholipid) | 24:1SMOH | 4.00E-04 | 2.0499 |
| Phosphatidylcholine diacyl C32:2 | Membrane Lipid (Phospholipid) | PC32:2AA | 2.00E-03 | 1.7815 |
| Phosphatidylcholine diacyl C36:6 | Membrane Lipid (Phospholipid) | PC36:6AA | 2.89E-05 | -1.9983 |
| Phosphatidylcholine diacyl C38:0 | Membrane Lipid (Phospholipid) | PC38:0AA | 5.57E-04 | 0.86156 |
| Phosphatidylcholine acyl-alkyl C40:6 | Membrane Lipid (Phospholipid) | PC40:6AE | 2.08E-02 | 1.0752 |
| Phosphatidylcholine diacyl C40:6 | Membrane Lipid (Phospholipid) | PC40:6AA | 1.43E-02 | 1.4696 |
| Phosphatidylcholine diacyl C40:1 | Membrane Lipid (Phospholipid) | PC40:1AA | 1.53E-03 | 1.2419 |
| L-Carnitine | Carnitine | C0 | 6.48E-09 | 12.385 |
| Acetylcarnitine | Carnitine | C2 | 3.01E-03 | 1.9981 |
| Butyrylcarnitine | Carnitine | C4 | 1.92E-06 | 5.1391 |
| Hydroxypropionylcarnitine | Carnitine | C3OH | 6.00E-03 | 1.6724 |
| Tiglylcarnitine | Carnitine | C5:1 | 1.45E-04 | 3.0902 |
| Valerylcarnitine | Carnitine | C5 | 5.67E-08 | 4.5343 |
| Hexenoylcarnitine | Carnitine | C6:1 | 1.57E-02 | 1.7366 |
| Hydroxyvalerylcarnitine (Methylmalonylcarnitine) | Carnitine | C5OH | 2.39E-03 | 1.4133 |
| Glutaconylcarnitine | Carnitine | C5:1DC | 8.66E-08 | 12.4 |
| Glutarylcarnitine (Hydroxyhexanoylcarnitine) | Carnitine | C5DC | 1.08E-04 | 2.7402 |
| Octanoylcarnitine | Carnitine | C8 | 8.84E-03 | 1.8606 |
| Methylglutarylcarnitine | Carnitine | C5MDC | 8.00E-03 | 1.1519 |
| Pimeloylcarnitine | Carnitine | C7DC | 1.59E-05 | 3.6368 |
| Decenoylcarnitine | Carnitine | C10:1 | 6.53E-04 | -1.4943 |
| Dodecenoylcarnitine | Carnitine | C12:1 | 2.21E-02 | 1.6728 |
| Dodecanoylcarnitine | Carnitine | C12 | 2.28E-07 | 4.4155 |
| Tetradecadienoylcarnitine | Carnitine | C14:2 | 1.36E-09 | 12.383 |
| Tetradecenoylcarnitine | Carnitine | C14:1 | 8.02E-08 | 3.5973 |
| Hydroxytetradecadienoylcarnitine | Carnitine | C14:2OH | 1.14E-02 | 1.9593 |
| Hydroxytetradecenoylcarnitine | Carnitine | C14:1OH | 1.19E-03 | 1.6567 |
| Hexadecenoylcarnitine | Carnitine | C16:1 | 5.32E-05 | 2.5668 |
| Hydroxyhexadecadienoylcarnitine | Carnitine | C16:2OH | 1.93E-02 | 1.7121 |
| Hydroxyoctadecenoylcarnitine | Carnitine | C18:1OH | 3.31E-04 | 3.218 |
1FC = Fold change, HRPmean/INFmean

## Slide 5
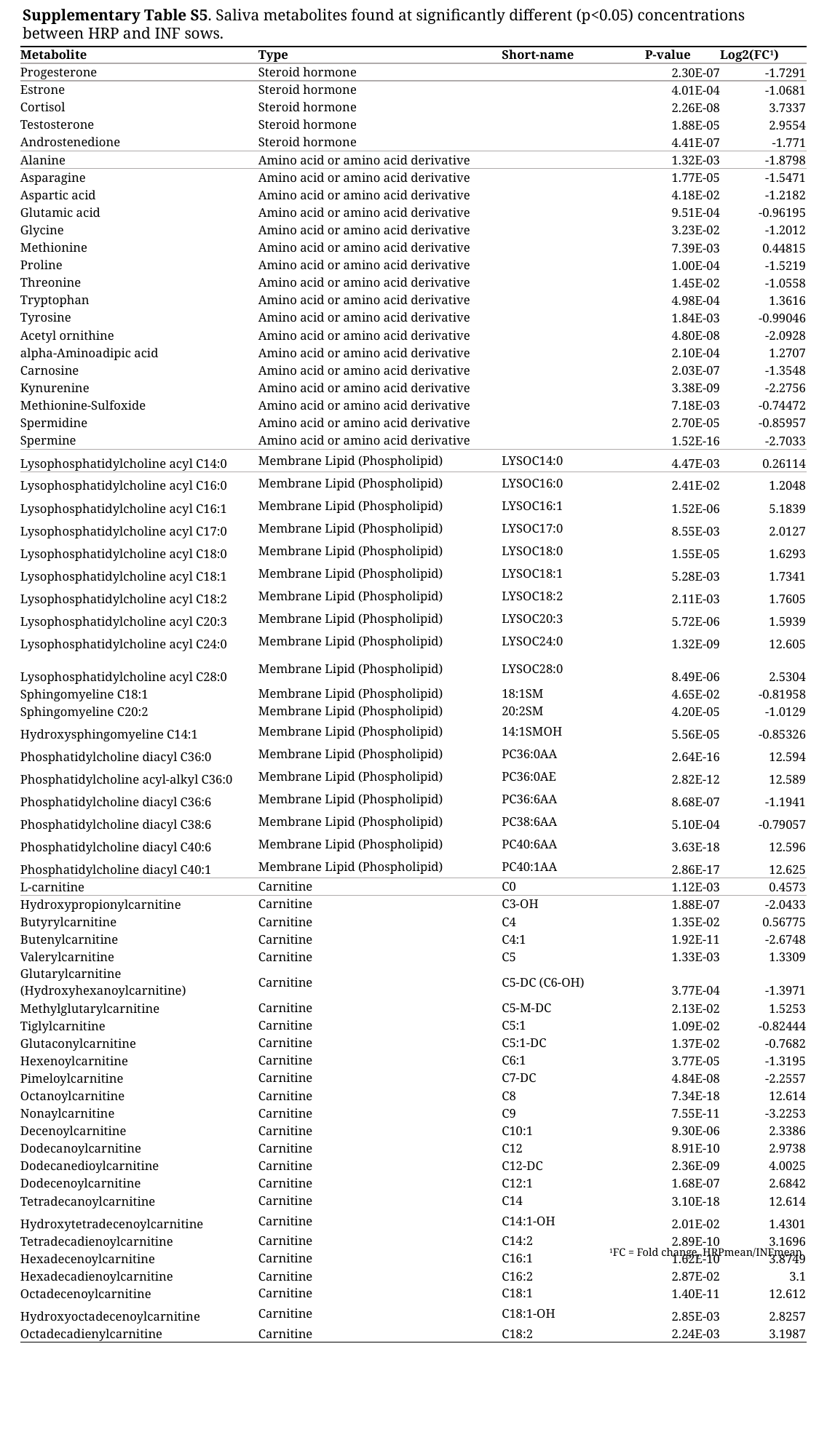

Supplementary Table S5. Saliva metabolites found at significantly different (p<0.05) concentrations between HRP and INF sows.
| Metabolite | Type | Short-name | P-value | Log2(FC1) |
| --- | --- | --- | --- | --- |
| Progesterone | Steroid hormone | | 2.30E-07 | -1.7291 |
| Estrone | Steroid hormone | | 4.01E-04 | -1.0681 |
| Cortisol | Steroid hormone | | 2.26E-08 | 3.7337 |
| Testosterone | Steroid hormone | | 1.88E-05 | 2.9554 |
| Androstenedione | Steroid hormone | | 4.41E-07 | -1.771 |
| Alanine | Amino acid or amino acid derivative | | 1.32E-03 | -1.8798 |
| Asparagine | Amino acid or amino acid derivative | | 1.77E-05 | -1.5471 |
| Aspartic acid | Amino acid or amino acid derivative | | 4.18E-02 | -1.2182 |
| Glutamic acid | Amino acid or amino acid derivative | | 9.51E-04 | -0.96195 |
| Glycine | Amino acid or amino acid derivative | | 3.23E-02 | -1.2012 |
| Methionine | Amino acid or amino acid derivative | | 7.39E-03 | 0.44815 |
| Proline | Amino acid or amino acid derivative | | 1.00E-04 | -1.5219 |
| Threonine | Amino acid or amino acid derivative | | 1.45E-02 | -1.0558 |
| Tryptophan | Amino acid or amino acid derivative | | 4.98E-04 | 1.3616 |
| Tyrosine | Amino acid or amino acid derivative | | 1.84E-03 | -0.99046 |
| Acetyl ornithine | Amino acid or amino acid derivative | | 4.80E-08 | -2.0928 |
| alpha-Aminoadipic acid | Amino acid or amino acid derivative | | 2.10E-04 | 1.2707 |
| Carnosine | Amino acid or amino acid derivative | | 2.03E-07 | -1.3548 |
| Kynurenine | Amino acid or amino acid derivative | | 3.38E-09 | -2.2756 |
| Methionine-Sulfoxide | Amino acid or amino acid derivative | | 7.18E-03 | -0.74472 |
| Spermidine | Amino acid or amino acid derivative | | 2.70E-05 | -0.85957 |
| Spermine | Amino acid or amino acid derivative | | 1.52E-16 | -2.7033 |
| Lysophosphatidylcholine acyl C14:0 | Membrane Lipid (Phospholipid) | LYSOC14:0 | 4.47E-03 | 0.26114 |
| Lysophosphatidylcholine acyl C16:0 | Membrane Lipid (Phospholipid) | LYSOC16:0 | 2.41E-02 | 1.2048 |
| Lysophosphatidylcholine acyl C16:1 | Membrane Lipid (Phospholipid) | LYSOC16:1 | 1.52E-06 | 5.1839 |
| Lysophosphatidylcholine acyl C17:0 | Membrane Lipid (Phospholipid) | LYSOC17:0 | 8.55E-03 | 2.0127 |
| Lysophosphatidylcholine acyl C18:0 | Membrane Lipid (Phospholipid) | LYSOC18:0 | 1.55E-05 | 1.6293 |
| Lysophosphatidylcholine acyl C18:1 | Membrane Lipid (Phospholipid) | LYSOC18:1 | 5.28E-03 | 1.7341 |
| Lysophosphatidylcholine acyl C18:2 | Membrane Lipid (Phospholipid) | LYSOC18:2 | 2.11E-03 | 1.7605 |
| Lysophosphatidylcholine acyl C20:3 | Membrane Lipid (Phospholipid) | LYSOC20:3 | 5.72E-06 | 1.5939 |
| Lysophosphatidylcholine acyl C24:0 | Membrane Lipid (Phospholipid) | LYSOC24:0 | 1.32E-09 | 12.605 |
| Lysophosphatidylcholine acyl C28:0 | Membrane Lipid (Phospholipid) | LYSOC28:0 | 8.49E-06 | 2.5304 |
| Sphingomyeline C18:1 | Membrane Lipid (Phospholipid) | 18:1SM | 4.65E-02 | -0.81958 |
| Sphingomyeline C20:2 | Membrane Lipid (Phospholipid) | 20:2SM | 4.20E-05 | -1.0129 |
| Hydroxysphingomyeline C14:1 | Membrane Lipid (Phospholipid) | 14:1SMOH | 5.56E-05 | -0.85326 |
| Phosphatidylcholine diacyl C36:0 | Membrane Lipid (Phospholipid) | PC36:0AA | 2.64E-16 | 12.594 |
| Phosphatidylcholine acyl-alkyl C36:0 | Membrane Lipid (Phospholipid) | PC36:0AE | 2.82E-12 | 12.589 |
| Phosphatidylcholine diacyl C36:6 | Membrane Lipid (Phospholipid) | PC36:6AA | 8.68E-07 | -1.1941 |
| Phosphatidylcholine diacyl C38:6 | Membrane Lipid (Phospholipid) | PC38:6AA | 5.10E-04 | -0.79057 |
| Phosphatidylcholine diacyl C40:6 | Membrane Lipid (Phospholipid) | PC40:6AA | 3.63E-18 | 12.596 |
| Phosphatidylcholine diacyl C40:1 | Membrane Lipid (Phospholipid) | PC40:1AA | 2.86E-17 | 12.625 |
| L-carnitine | Carnitine | C0 | 1.12E-03 | 0.4573 |
| Hydroxypropionylcarnitine | Carnitine | C3-OH | 1.88E-07 | -2.0433 |
| Butyrylcarnitine | Carnitine | C4 | 1.35E-02 | 0.56775 |
| Butenylcarnitine | Carnitine | C4:1 | 1.92E-11 | -2.6748 |
| Valerylcarnitine | Carnitine | C5 | 1.33E-03 | 1.3309 |
| Glutarylcarnitine (Hydroxyhexanoylcarnitine) | Carnitine | C5-DC (C6-OH) | 3.77E-04 | -1.3971 |
| Methylglutarylcarnitine | Carnitine | C5-M-DC | 2.13E-02 | 1.5253 |
| Tiglylcarnitine | Carnitine | C5:1 | 1.09E-02 | -0.82444 |
| Glutaconylcarnitine | Carnitine | C5:1-DC | 1.37E-02 | -0.7682 |
| Hexenoylcarnitine | Carnitine | C6:1 | 3.77E-05 | -1.3195 |
| Pimeloylcarnitine | Carnitine | C7-DC | 4.84E-08 | -2.2557 |
| Octanoylcarnitine | Carnitine | C8 | 7.34E-18 | 12.614 |
| Nonaylcarnitine | Carnitine | C9 | 7.55E-11 | -3.2253 |
| Decenoylcarnitine | Carnitine | C10:1 | 9.30E-06 | 2.3386 |
| Dodecanoylcarnitine | Carnitine | C12 | 8.91E-10 | 2.9738 |
| Dodecanedioylcarnitine | Carnitine | C12-DC | 2.36E-09 | 4.0025 |
| Dodecenoylcarnitine | Carnitine | C12:1 | 1.68E-07 | 2.6842 |
| Tetradecanoylcarnitine | Carnitine | C14 | 3.10E-18 | 12.614 |
| Hydroxytetradecenoylcarnitine | Carnitine | C14:1-OH | 2.01E-02 | 1.4301 |
| Tetradecadienoylcarnitine | Carnitine | C14:2 | 2.89E-10 | 3.1696 |
| Hexadecenoylcarnitine | Carnitine | C16:1 | 1.62E-10 | 3.8749 |
| Hexadecadienoylcarnitine | Carnitine | C16:2 | 2.87E-02 | 3.1 |
| Octadecenoylcarnitine | Carnitine | C18:1 | 1.40E-11 | 12.612 |
| Hydroxyoctadecenoylcarnitine | Carnitine | C18:1-OH | 2.85E-03 | 2.8257 |
| Octadecadienylcarnitine | Carnitine | C18:2 | 2.24E-03 | 3.1987 |
1FC = Fold change, HRPmean/INFmean

## Slide 6
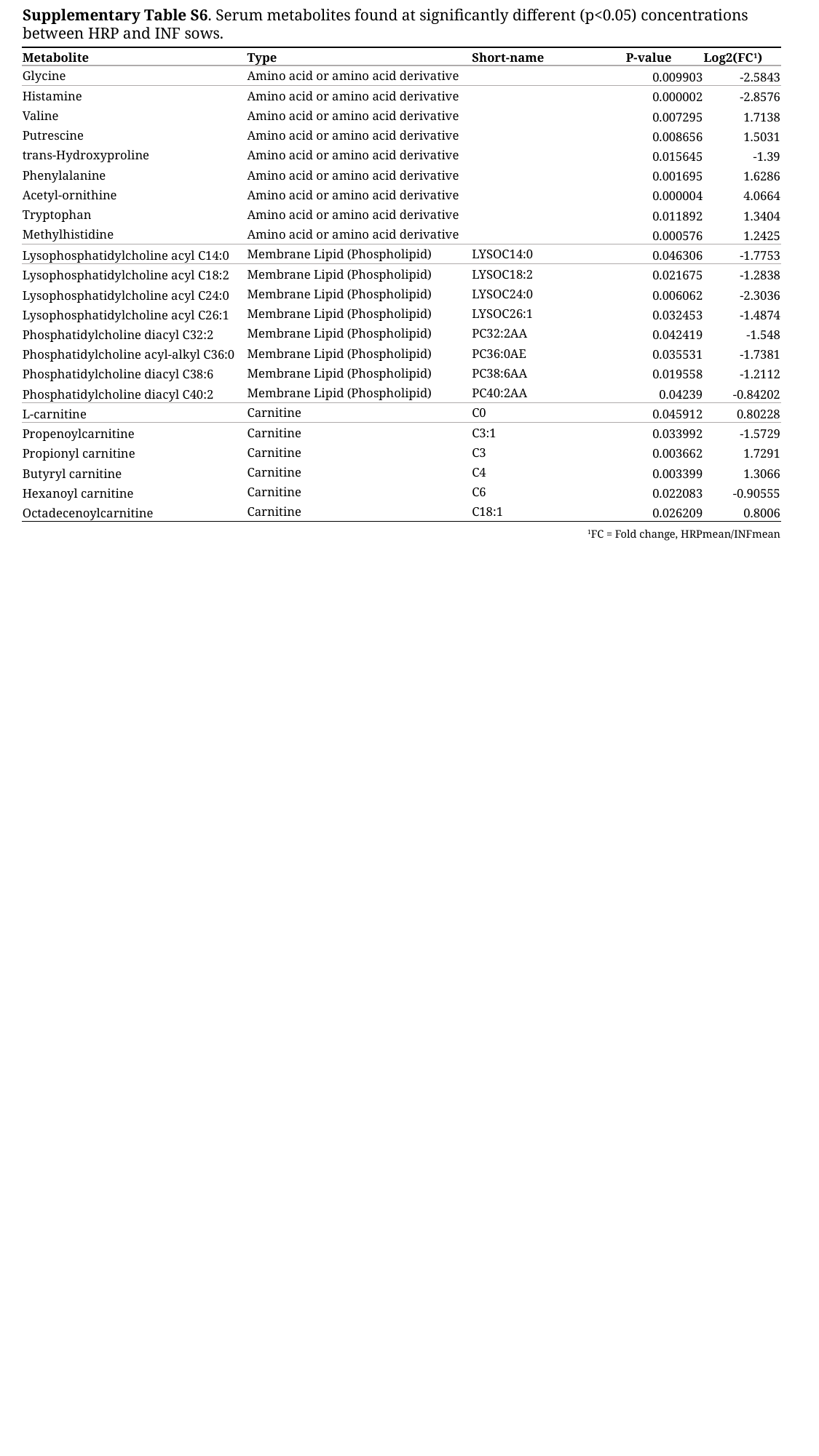

Supplementary Table S6. Serum metabolites found at significantly different (p<0.05) concentrations between HRP and INF sows.
| Metabolite | Type | Short-name | P-value | Log2(FC1) |
| --- | --- | --- | --- | --- |
| Glycine | Amino acid or amino acid derivative | | 0.009903 | -2.5843 |
| Histamine | Amino acid or amino acid derivative | | 0.000002 | -2.8576 |
| Valine | Amino acid or amino acid derivative | | 0.007295 | 1.7138 |
| Putrescine | Amino acid or amino acid derivative | | 0.008656 | 1.5031 |
| trans-Hydroxyproline | Amino acid or amino acid derivative | | 0.015645 | -1.39 |
| Phenylalanine | Amino acid or amino acid derivative | | 0.001695 | 1.6286 |
| Acetyl-ornithine | Amino acid or amino acid derivative | | 0.000004 | 4.0664 |
| Tryptophan | Amino acid or amino acid derivative | | 0.011892 | 1.3404 |
| Methylhistidine | Amino acid or amino acid derivative | | 0.000576 | 1.2425 |
| Lysophosphatidylcholine acyl C14:0 | Membrane Lipid (Phospholipid) | LYSOC14:0 | 0.046306 | -1.7753 |
| Lysophosphatidylcholine acyl C18:2 | Membrane Lipid (Phospholipid) | LYSOC18:2 | 0.021675 | -1.2838 |
| Lysophosphatidylcholine acyl C24:0 | Membrane Lipid (Phospholipid) | LYSOC24:0 | 0.006062 | -2.3036 |
| Lysophosphatidylcholine acyl C26:1 | Membrane Lipid (Phospholipid) | LYSOC26:1 | 0.032453 | -1.4874 |
| Phosphatidylcholine diacyl C32:2 | Membrane Lipid (Phospholipid) | PC32:2AA | 0.042419 | -1.548 |
| Phosphatidylcholine acyl-alkyl C36:0 | Membrane Lipid (Phospholipid) | PC36:0AE | 0.035531 | -1.7381 |
| Phosphatidylcholine diacyl C38:6 | Membrane Lipid (Phospholipid) | PC38:6AA | 0.019558 | -1.2112 |
| Phosphatidylcholine diacyl C40:2 | Membrane Lipid (Phospholipid) | PC40:2AA | 0.04239 | -0.84202 |
| L-carnitine | Carnitine | C0 | 0.045912 | 0.80228 |
| Propenoylcarnitine | Carnitine | C3:1 | 0.033992 | -1.5729 |
| Propionyl carnitine | Carnitine | C3 | 0.003662 | 1.7291 |
| Butyryl carnitine | Carnitine | C4 | 0.003399 | 1.3066 |
| Hexanoyl carnitine | Carnitine | C6 | 0.022083 | -0.90555 |
| Octadecenoylcarnitine | Carnitine | C18:1 | 0.026209 | 0.8006 |
1FC = Fold change, HRPmean/INFmean
